# An Enduring Role for Hippocampal Pattern Completion in Addition to an Emergent Nonhippocampal Contribution to Holistic Episodic Retrieval after a 24 h Delay

**DOI:** 10.1523/JNEUROSCI.1740-23.2024

**Published:** 2024-03-25

**Authors:** Bárður H. Joensen, Jennifer E. Ashton, Sam C. Berens, M. Gareth Gaskell, Aidan J. Horner

**Affiliations:** ^1^Department of Psychology, Lund University, Lund 221 00, Sweden; ^2^Department of Psychology, University of York, York YO10 5DD, United Kingdom; ^3^School of Psychology, University of Sussex, Falmer BN1 9QH, United Kingdom; ^4^York Biomedical Research Institute, University of York, York YO10 5NG, United Kingdom

**Keywords:** episodic memory, fMRI, hippocampus, systems consolidation

## Abstract

Episodic memory retrieval is associated with the holistic neocortical reinstatement of all event information, an effect driven by hippocampal pattern completion. However, whether holistic reinstatement occurs, and whether hippocampal pattern completion continues to drive reinstatement, after a period of consolidation is unclear. Theories of systems consolidation predict either a time-variant or time-invariant role of the hippocampus in the holistic retrieval of episodic events. Here, we assessed whether episodic events continue to be reinstated holistically and whether hippocampal pattern completion continues to facilitate holistic reinstatement following a period of consolidation. Female and male human participants learned “events” that comprised multiple overlapping pairs of event elements (e.g., person–location, object–location, location–person). Importantly, encoding occurred either immediately before or 24 h before retrieval. Using fMRI during the retrieval of events, we show evidence for holistic reinstatement, as well as a correlation between reinstatement and hippocampal pattern completion, regardless of whether retrieval occurred immediately or 24 h after encoding. Thus, hippocampal pattern completion continues to contribute to holistic reinstatement after a delay. However, our results also revealed that some holistic reinstatement can occur without evidence for a corresponding signature of hippocampal pattern completion after a delay (but not immediately after encoding). We therefore show that hippocampal pattern completion, in addition to a nonhippocampal process, has a role in holistic reinstatement following a period of consolidation. Our results point to a consolidation process where the hippocampus and neocortex may work in an additive, rather than compensatory, manner to support episodic memory retrieval.

## Significance Statement

The hippocampus plays a critical role in the recollection of episodic events. However, its role in episodic memory retrieval over time is still debated with theories of systems consolidation predicting both time-limited and time-invariant roles. We used fMRI and an experimental assay of holistic episodic memory retrieval to assess hippocampal pattern completion and its relationship to neocortical reinstatement across delay. We provide evidence that hippocampal pattern completion is associated with neocortical reinstatement following delay but that some reinstatement can occur without a corresponding signature of hippocampal pattern completion. Our results suggest that hippocampal pattern completion, in addition to an emergent nonhippocampal process, may play an additive role in the recollection of episodic memories following a period of consolidation.

## Introduction

The recollection of episodic events is holistic in nature (Tulving, 1983; Yonelinas, 1994), underpinned by a hippocampal pattern completion process (Marr, 1971; Hopfield, 1982; Treves and Rolls, 1992; McClelland et al., 1995; Norman and O’Reilly, 2003) that drives the reinstatement of event information in the neocortex (Wheeler et al., 2000; Woodruff et al., 2005; Johnson et al., 2009).

Evidence for hippocampal pattern completion and holistic neocortical reinstatement has been shown immediately following encoding (Horner et al., 2015; Grande et al., 2019). However, we do not yet know whether this relationship changes following a delay. This is a critical question as standard theories of consolidation predict a time-limited role for the hippocampus (McClelland et al., 1995; Squire and Alvarez, 1995), while others argue that the hippocampus is always required for episodic retrieval (Nadel and Moscovitch, 1997; Winocur and Moscovitch, 2011; Sekeres et al., 2018).

There is evidence for decreases in (Niki and Luo, 2002; Takashima et al., 2006; Takashima et al., 2009) and sustained (Maguire et al., 2001; Ryan et al., 2001; Addis et al., 2004; Gilboa et al., 2004; Bonnici et al., 2012) hippocampal activity during retrieval following a delay (see also Ritchey et al., 2015). Critically, most previous studies have compared hippocampal activity (or multivariate patterns) at immediate and delayed retrieval and therefore have not directly assessed whether hippocampal pattern completion continues to mediate the reinstatement of episodic events in the neocortex. Importantly, changes in univariate activity over time could be driven by different mechanisms not related to systems consolidation, such as differential rates of forgetting for independent neocortical and hippocampal representations formed at encoding (Sekeres et al., 2016; Fisher and Radvansky, 2018; Andermane et al., 2021). Thus, by directly assessing the relationship between hippocampal pattern completion and neocortical reinstatement across delay, we can more directly assess the mechanistic predictions of systems consolidation theories.

Here, we used an experimental assay of episodic memory (Horner and Burgess, 2014; Horner et al., 2015) that allowed us to assess the delay-(in)variant role of hippocampal pattern completion in neocortical reinstatement. Across two encoding sessions, separated for ∼24 h, participants learned three-element events. Events were learned over two or three separate encoding trials, with each trial presenting one of three possible pairs for an event. Events had different associative structures: “closed-loops” where all three pairs were encoded and “open-loops” where only two of the three pairs were encoded. At retrieval, we tested each of the learned pairs from each event, with half of the events learned ∼24 h before retrieval and the other half learned immediately before retrieval.

We identified neocortical regions that showed differences in the BOLD activity between elements that were the cue or target of retrieval and those that were nontargets. Greater activity for closed- versus open-loops in nontarget regions was then used as a marker of holistic neocortical reinstatement (Horner et al., 2015). Next, we correlated nontarget neocortical reinstatement with a signature of hippocampal pattern completion (closed- vs open-loop hippocampal activity), for events encoded immediately and ∼24 h earlier. We used a general linear mixed-effects model to assess both the slope and the intercept of the relationship between the hippocampal contrast and neocortical reinstatement.

A slope greater than zero would indicate a positive correlation, consistent with hippocampal pattern completion contributing to holistic neocortical reinstatement. If the steepness of the slope is consistent across delay, this would indicate a continued role for hippocampal pattern completion, while a shallower (or zero) slope in the delay relative to immediate condition would suggest a time-limited role. Critically, in the presence of a positive slope, a zero intercept would suggest that holistic reinstatement only occurs in the presence of hippocampal pattern completion. An intercept greater than zero would indicate that some holistic reinstatement can occur without a corresponding signature of pattern completion. This would point to an emergent nonhippocampal contribution that is additive (rather than compensatory) to the role of hippocampal pattern completion in holistic reinstatement.

## Materials and Methods

### Participants

An effect size *d* = 0.67 for detecting a significant nontarget reinstatement effect was calculated from previous work (Horner et al., 2015) with *n* = 26. Cohen’s *d* was calculated from the reported *t*-statistic divided by the square root of the within-subject sample size (Lakens, 2013). As forgetting is expected to occur when retrieval follows a ∼24 h delay, retrieval accuracy is expected to be lower than the finding reported in Horner et al. (2015). Pilot data collected with *n* = 12 revealed ∼14% decrease in retrieval accuracy following a 24 h delay, leading to an adjusted effect size *d* = 0.62. Using G*Power (Faul et al., 2009) and the adjusted effect size *d* = 0.62, we estimated that a sample size of *n* = 30 would be required to detect a significant nontarget reinstatement effect, if one is present, at a power of 0.90 and *α* = 0.05.

Given that previously reported effect sizes are likely to be overestimates of the true effect size, and because we wanted to compare neocortical reinstatement as well as hippocampal–neocortical correlations between two conditions, we aimed to obtain 50 useable datasets (upper limit set by resource constraints). An initial sample of *n* = 30 was collected and analyzed (due to time constraints in relation to submitting a PhD thesis; reported in Joensen, 2019) before the final sample was collected.

Fifty-seven right-handed participants were recruited from the University of York student population. All participants gave written informed consent and took part in exchange for course credit or monetary compensation. Participants had either normal or correct-to-normal vision and reported no history of neurological or psychiatric illness. Data from seven participants could not be included in the final sample due to motion-related artifacts in the imaging data (*n* = 1), scanner faults resulting in no acquired imaging data (*n* = 2), or below chance memory performance (accuracy <∼16.7%; *n* = 4). Accordingly, the analyses included 50 participants (39 female, 11 male) with a mean age of 21.6 years ± 4.43 SD. The experiment was approved by the York Neuroimaging Centre ethics committee, University of York, United Kingdom.

### Materials

The stimuli consisted of 72 locations (e.g., kitchen), 72 famous people (e.g., Barack Obama), and 72 common objects (e.g., hammer). From these, 72 random location–person–object events were generated for each participant. Note that we used the term “event” to refer to the three elements (location, person, object) that were assigned to the same associative structure (closed- or open-loop), leaving aside any considerations as to how these relate to real-world events (Horner et al., 2015).

Each of the 72 events was assigned to the within-subject conditions of loop (open-loop or closed-loop) and delay (delay or no-delay). Thus, 18 different events were randomly assigned to either (1) open-loop, delay; (2) closed-loop, delay; (3) open-loop, no-delay; or (4) closed-loop, no-delay. For closed-loops, all three possible pairwise associations for each event were encoded, while for open-loops, only two out of the three possible pairwise associations were learnt. Events were never presented together at encoding or retrieval; only specific pairwise associations were encoded and retrieved for each event, depending on whether they were open- or closed-loop. Note that open-loops are equated to closed-loops in the number of elements, but not in the number of pairs. We have previously shown (Horner and Burgess, 2014; Horner et al., 2015) that dependency is not seen for open-loops when three overlapping pairs are encoded as an associated chain (e.g., kitchen–hammer, kitchen–Barack Obama, Barack Obama–dog), controlling for the number of associations (but not the number of elements). Any differences in retrieval between the two conditions in the current experiment are therefore unlikely to be driven by differences in the number of associations.

### Procedure

The experiment consisted of two encoding sessions and a single retrieval session. Both encoding sessions took place outside the scanner and were separated by ∼24 h (M ± SD* *= 23 h, 54 min ± 130 min), including overnight sleep. The retrieval session took place inside the scanner immediately after the second encoding session.

#### Encoding

**Figure 1.**
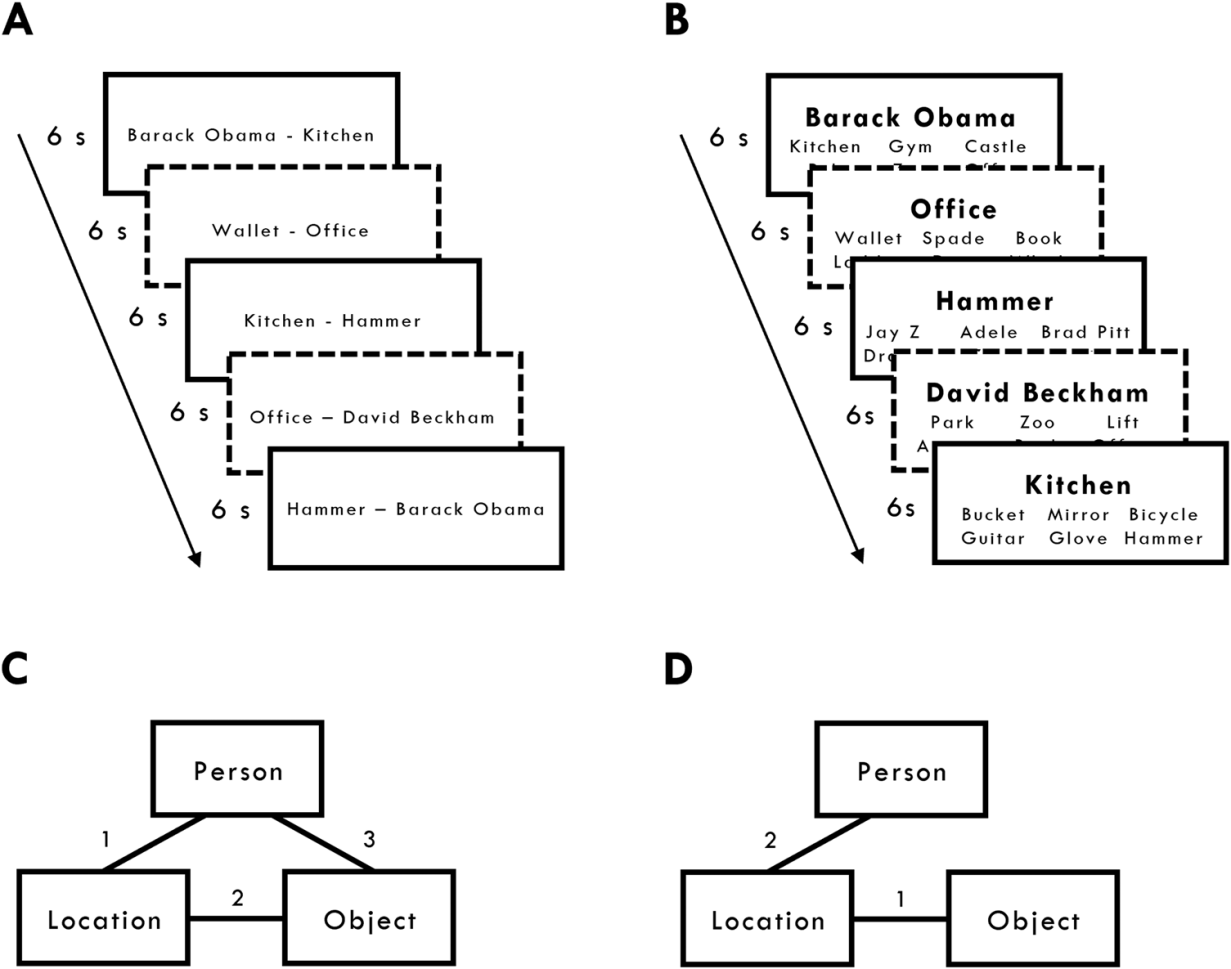
Experimental design. ***A***, Encoding. Participants saw multiple pairwise associations. They imagined each association “interacting in a meaningful way as vividly as possible” for 6 s. Each association was preceded by a 500 ms fixation and followed by a 500 ms blank screen. Participants encoded open- and closed-loop pairwise associations in an intermixed manner. In the open-loop condition, participants did not encode the third and final association (e.g., wallet–Beckham; see ***D***). Solid and dotted lines were not presented but highlighted closed- (solid lines) and open-loops (dotted lines). ***B***, Retrieval. Participants were presented with a single cue and required to retrieve one of the other elements from the same event from among the five foils (elements of the same type from other events) in 6 s. Each cued-recognition trial was preceded by a 1 s fixation and followed by a 1 s blank screen. ***C***, The associative structures of closed-loops with example encoding order for three pairwise associations (numbers 1–3). ***D***, The associative structure of open-loops with example encoding order for the two pairwise associations (numbers 1–2). The third and final association (i.e., person–object in this example) was not shown to the participants.

During encoding, participants were presented with specific pairwise associations from each of the 72 events. Participants learned one pairwise association per encoding trial. All pairwise associations were presented on a computer screen as words, with one item to the left and one item to the right of fixation (Fig. 1*A*). The left/right assignment was randomly chosen on each trial. The words remained on screen for 6 s. Participants were instructed to imagine, as vividly as possible, the elements interacting in a meaningful way for the full 6 s. Each word-pair presentation was preceded by a 500 ms fixation and followed by a 500 ms blank screen. For 36 out of the 72 events, participants encoded all three possible pairwise associations; forming closed-loops (Fig. 1*C*). For the remaining 36 triplets, participants encoded two out of the three possible pairwise associations; forming open-loops (Fig. 1*D*). Eighteen closed- and 18 open-loops were randomly assigned to each of the encoding sessions, resulting in a total of 90 encoding trials per encoding session (i.e., 54 closed-loop and 36 open-loop trials per session). Participants were not told that any pairwise associations would overlap with each other at encoding nor were they told about the open- and closed-loop structures.

Each encoding session consisted of three blocks of 18, 36, and 36 trials, respectively, with one pairwise association from each event presented during each block. Each encoding session contained novel events for the open- and closed-loop conditions that were not presented in the other encoding session. During the first encoding block, only pairwise associations for closed-loops were presented. This ensured that the duration between encoding the last pairwise association and retrieval was consistent for closed- and open-loops from each encoding session. In blocks 2 and 3, the open- and closed-loop associations were randomly presented in an intermixed manner. The presentation order was randomized for each block. A break of 10 s followed every 18 encoding trials.

Each open-loop consisted of a common element (e.g., if participants encoded location–person, and location–object pairs, then location would be the common element). For each encoding session, 6 out of the total 18 open-loops were pseudorandomly assigned to one of the three possible common elements (i.e., location, people, or objects). The presentation order for the open-loops across encoding blocks 2 and 3 was (1) person–location, location–object; (2) location–object, object–person; and (3) object–person, person–location. The presentation order for the closed-loops across the three encoding blocks was (1) person–location, location–object, object–person; (2) location–object, object–person, person–location; and (3) object–person, person–location, location–object. These counterbalanced orders ensured that the first two encoding trials for open-loops and closed-loops were identical in structure, such that closed-loops only differed in relation to the presentation of a third encoding trial.

#### Retrieval

During retrieval, participants performed a six-alternative forced-choice cued-recognition task. On each trial, a cue and six potential targets were presented simultaneously on the screen. The cue was presented in the middle of the screen with the six possible targets; one target and five foils form the same category (e.g., if the target was hammer, the five foils would be other randomly selected objects from the other events, regardless of closed- vs open-loop or delay vs no-delay status) presented in two rows of three below the cue (Fig. 1*B*). Participants had a maximum of 6 s to respond with a button response that corresponded to the target position on the screen and were instructed to be as accurate as possible in the time given. The position of the correct target was randomly selected on each retrieval trial. The cue and six targets were presented until a response was made or when the maximum 6 s limit was reached (M response times* ± *SD = 2.86 s ± 0.42 s). Missing responses (i.e., responses that fell outside the 6 s response window) were treated as incorrect trials for both accuracy and dependency (M percentage of missing responses* ± *SD = 3.54% ± 3.22%). A 2 × 2 (loop × delay) ANOVA, where the dependent variable was the proportion of missing responses, showed no significant effects (*F*s < 0.37). Thus, any differences in dependency across conditions are unlikely to be caused by assuming missing responses would have been incorrect. Note also that due to the six-alternative forced-choice recognition test, the chance of guessing correctly was relatively low (∼16.7%).

Each encoded pairwise association (regardless of delay or loop status) was tested in both directions (e.g., cue, location; target, person; cue, person; target, location) across two scanning runs (total retrieval trials, 360; 180 trials per run). For the open-loops, we only tested the pairwise associations that were directly encoded—and not those that could potentially be inferred from overlapping nature of pairwise associations (Shohamy and Wagner, 2008; Zeithamova et al., 2012)—resulting in four retrieval trials per open-loop compared with six retrieval trials per closed-loop. The presentation order was optimized to measure the univariate BOLD activity in each of the four within-subject experimental conditions (i.e., open-loop, delay; open-loop, no-delay; closed-loop, delay; closed-loop, no-delay; optimization algorithm available at https://osf.io/eh78w/). To avoid adaptation effects, cue–target associations from the same closed- or open-loop were never presented on successive trials, and each block contained 18 null trials that each lasted 6 s. In each retrieval block (or scan run), cue–target associations belonging to nine closed- and open-loops encoded during the first encoding session and nine closed- and nine open-loops encoded immediately before retrieval were tested in a random order, making a total of 180 retrieval trials, in addition to the 18 null trials. Each trial was preceded by a 1 s fixation and followed by a 1 s blank screen.

### Modeling retrieval dependency

To model retrieval dependency, we created 2 × 2 contingency tables for the observed data for each participant to assess the proportion of joint retrieval and joint nonretrieval between the retrieval of two elements (e.g., person, object) when cued by a common element (e.g., location; A_B_A_C_) and between the retrieval of a common element (e.g., location) when cued by the other two elements from the same (e.g., person, object; B_A_C_A_). For the closed-loops, six contingency tables were constructed (as we test all three pairwise associations in both directions), and for the open-loops, four contingency tables were constructed (as we only tested the two directly encoded pairwise associations in both directions).

We also constructed contingency tables that estimate the number of trials that would fall into each of the four cells if the retrieval of pairwise associations within an open- or closed-loop was independent. This independent model assumes that pairwise associations from a given event are retrieved independently of one another—that is, if a participant retrieved one pairwise association from an event (un)successfully, this does not predict the participants ability to (un)successfully retrieve another pairwise association from the same event.

The 2 × 2 contingency tables for the data show the number of events that fall within the four cells (i.e., for the A_B_A_C_ analysis, both A_B_ and A_C_ correct, A_B_ incorrect and A_C_ correct; A_B_ correct and A_C_ incorrect; and both A_B_ and A_C_ incorrect, where A_B_ = cue with location (A) and retrieve person (B) and similarly for A_C_, where C refers to object). The table for the independent model (Table 1) shows the predicted number of events that fall in the four cells, given a participant’s overall level of accuracy, when the retrieval of within-event associations is assumed to be independent.

**Table 1.** Contingency table for the independent model for correct and incorrect retrieval, over *N* events (*i* = 1 to *N*), for elements B and C when cued by A

Retrieval of element C	Retrieval of element B	
	Correct $\left(P_{\mathrm{AB}}\right)$	Incorrect $\left(1\;\;-\;P_{\mathrm{AB}}\right)$
Correct $\left(P_{\mathrm{AC}}\right)$	$\overset{N}{\underset{i\;=1}{\sum }}=P_{AB_{i}}\;P_{AC_{i}}$	$\overset{N}{\underset{i\;=1}{\sum }}=P_{AC_{i}}\;(1\;-\;P_{AB_{i}})$
Incorrect $(1-\;P_{\mathrm{AC}})$	$\overset{N}{\underset{i=1}{\sum }}=P_{AB_{i}}\;(1\;\;-\;P_{AC_{i}})$	$\overset{N}{\underset{i\;=1}{\sum }}=(1\;-\;P_{AB_{i}})\;(1\;-\;P_{AC_{i}})$

For a given participant, the proportion of correct retrievals of, for instance, element B when cued by A is denoted by $P_{\mathrm{AB}}\;$ (i.e., the mean performance for B when cued by A across all events). For the independent model, when cued by A, the probability of (1) correctly retrieving B and C (across all events) is equal to $P_{\mathrm{AB}}\;P_{\mathrm{AC}}$; (2) correctly retrieving B but not C is equal to $P_{\mathrm{AB}}\;(1\;-\;P_{\mathrm{AC}})$; (3) correctly retrieving C but not B is equal to $P_{\mathrm{AC}}\;(1\;-\;P_{\mathrm{AB}})\;$; and (4) incorrectly retrieving both B and C is equal to $(1\;\;-\;P_{\mathrm{AB}})\;(1\;-\;P_{\mathrm{AC}})$.

Once the contingency tables for the data and the independent model were constructed, the proportion of joint retrieval and joint nonretrieval were calculated for each contingency table separately, by summing the leading diagonal cells and dividing by the total number of events (i.e., the proportion of events where two overlapping pairwise associations within an event are both retrieved either correctly or incorrectly). This measure was then averaged across the six or four contingency tables (dependent on whether the tables related to open- or closed-loops) to provide a single measure of the proportion of joint retrieval and nonretrieval for the data and independent model separately. For brevity, we refer to this measure as the “proportion of joint retrieval,” but note that it includes the proportion of both joint retrieval and joint nonretrieval.

This proportion of joint retrieval measure scales with accuracy. We therefore compare this measure in the data relative to the independent model. As such, the independent model serves as a lower bound that can be compared with the proportion of joint retrieval in the data. We therefore have a single measure of “retrieval dependency” (i.e., the difference between the proportion of joint retrieval in the data relative to the independent model) for each participant and condition.

### Behavioral analyses

A 2 × 2 (loop × delay) ANOVA was used for the behavioral analysis of retrieval accuracy and retrieval dependency, with the within-subject factor loop referring to whether the events formed closed- or open-loops and delay referring to whether closed- or open-loops were encoded immediately (no-delay) or 24 h prior to retrieval (delay). For the analysis of retrieval dependency, we also report one sample *t* tests comparing retrieval dependency in each condition with zero. Retrieval dependency significantly greater than zero provides evidence for the presence of dependency. Alpha was set to 0.05 (two-tailed) for all statistical tests. For each ANOVA, a $\eta_{p}^{2}$ effect size is reported, and for *t* tests we report a Cohen’s *d* effect size reflecting the mean difference between conditions divided by the standard deviation of the difference scores. All analyses were conducted using JASP (JASP Team, 2021).

### fMRI acquisition

All functional and structural volumes were acquired on a 3 T Siemens MAGNETOM Prisma scanner equipped with a 64-channel phase array head coil. T2*-weighted slices were acquired with echo-planar imaging (EPI). Forty-eight axial slices (∼0° tilt to the AC–PC lines) per volume were acquired in an interleaved order with the following parameters: acquisition matrix, 64 × 64; repetition time, 1,200 ms; echo time, 26 ms; flip angle, 75°; slice thickness, 3 mm; in-plane resolution, 3 × 3 mm; and multiband acceleration factor, 2. To allow for T1 equilibrium, we acquired the first three EPI volumes prior to the task and then discarded them. As the retrieval phase varied in length across participants (as each cued-recognition trial was self-paced, up to a maximum of 6 s per trial), the number of acquired volumes differed across participants. Note that the two retrieval blocks corresponded to two separate functional scanning runs. The mean number of volumes acquired during retrieval was 796.80 (range, 621–955) and 774.04 (range, 616–942) for the first and second retrieval blocks, respectively. A field map was acquired to allow for the correction of geometric distortions due to field inhomogeneities. For the purpose of coregistration and image normalization, a whole-brain T1-weighted structural scan was acquired with a 1 mm^3^ resolution using a magnetization-prepared rapid gradient echo pulse sequence.

### fMRI analyses

#### Preprocessing

Image preprocessing was performed in SPM12. EPI images were corrected for field inhomogeneity based on geometric distortions and spatially realigned to the first image of the time series. EPI images were spatially normalized to MNI space with transformation parameters derived from warping each participant’s structural image to a T1-weighted average template image (using the DARTEL toolbox; Ashburner, 2007). EPI images were spatially smoothed with an isotropic 8 mm FWHM Gaussian kernel prior to analysis.

#### General analysis

At the first level, BOLD activity was modeled by a boxcar function from cue/target onset to the time a response was made (up to a maximum of 6 s). The predicted BOLD response was then convolved with a canonical hemodynamic response function to produce regressors of interest. In addition to the main regressors of interest, all first-level models included a set of nuisance regressors: six movement parameters, their first-order derivatives, and volume exclusion regressors censoring periods of excessive motion. The volume exclusion regressors were defined as volumes where the movement derivatives exceeded 1.5 mm translation (i.e., 0.5 × slice thickness) or 1° rotation (M number of excluded volumes ± SD = 0.79 ± 3.60). Parameter estimates for each regressor of interest were included in the second-level analysis to identify consistent effects across participants. Unless otherwise stated, all second-level models explicitly modeled subject effects. All effects reported outside the hippocampus are *p*_FWE_ < 0.05 family-wise error (FWE) whole-brain corrected (cluster size >30 voxels). We also performed small-volume corrected (SVC) analyses in the hippocampus given the a priori predictions related to this brain region. This mask for SVC was created using the WFU PickAtlas toolbox (Maldjian et al., 2003) with the hippocampus defined from the Automated Anatomical Labeling atlas (Tzourio-Mazoyer et al., 2002).

#### Neocortical reinstatement

The first-level model included 24 regressors that related to all cue–target pairs (i.e., person–location, person–object, location–person, location–object, object–person, and object–location) for each of the four experimental conditions (open vs closed and delay vs no-delay). At the second level, regions that showed greater BOLD response to cueing and retrieving each element type (i.e., cue/target), relative to when an element type was not cued or required to be retrieved (i.e., nontarget), collapsed across the four experimental conditions were identified. This revealed three separate regions of interest across the three element types (Table 3). For each region of interest—defined as a 9-mm-radius sphere centered on the peak coordinate—BOLD responses for each individual across all 24 regressors were extracted. The BOLD response when the element associated with a given region was either the cue, target, or nontarget across the four experimental conditions was then calculated. As such, estimates of the BOLD response across 12 conditions (i.e., cue, target, and nontarget × closed- and open-loop × delay and no-delay) were obtained for each of the three regions of interest.

#### Nontarget reinstatement and correlated activity

The first-level model included four regressors corresponding to trials from each of the four experimental conditions (i.e., open-loop, delay; closed-loop, delay; open-loop, no-delay; and closed-loop, no-delay). At the second level, two separate random-effects models were created, one for the delay and one for the no-delay condition. Each model included a single regressor corresponding to the contrast between closed- and open-loops (i.e., open-loop vs closed-loops for the respective delay or no-delay condition). This was calculated by taking the mean difference in BOLD response between the closed- and open-loop conditions for the nontarget element across the three regions of interest. Given the prior hypothesis regarding the hippocampus, statistical effects in the bilateral hippocampus are *p* < 0.05 FWE SVC within a bilateral hippocampal mask.

### Data availability

Second-level data and analyses are available on the Open Science Framework (https://osf.io/hk79v/).

## Results

### Behavior

#### Retrieval accuracy

Memory performance (proportion correct) across each of the four experimental conditions is presented in Table 2.

**Table 2.** Mean proportion correct (and standard deviations) and mean proportion of joint retrieval (and standard deviations) for the data and independent model at no-delay (i.e., encoded immediately prior to retrieval delay) and delay (i.e., encoded 24 h prior to retrieval) for closed- and open-loops

Delay	Loop	Proportion correct	Proportion of joint retrieval	
			Data	Independent model
No-delay	Open	0.62 (0.19)	0.61 (0.14)	0.59 (0.12)
	Closed	0.68 (0.21)	0.69 (0.14)	0.65 (0.14)
Delay	Open	0.50 (0.19)	0.56 (0.13)	0.55 (0.11)
	Closed	0.61 (0.21)	0.67 (0.12)	0.61 (0.13)

A 2 × 2 (loop × delay) ANOVA revealed a significant effect of loop (*F*_(1,49)_ = 37.72; *p* < 0.001; $\eta_{p}^{2}$*^ ^*= 0.44) and delay (*F*_(1,49)_ = 45.74; *p* < 0.001; $\eta_{p}^{2}$*^ ^*= 0.48), with greater memory for closed-loops, relative to open-loops, and for pairs retrieved immediately after encoding, compared with those retrieved following a 24 h delay. The ANOVA also revealed a significant interaction between these factors (*F*_(1,49)_ = 10.78; *p* = 0.002; $\eta_{p}^{2}$ = 0.18), with a greater difference in memory between closed- and open-loops at delay relative to no-delay. This is consistent with the finding reported in Joensen et al. (2020).

#### Retrieval dependency

**Figure 2.**
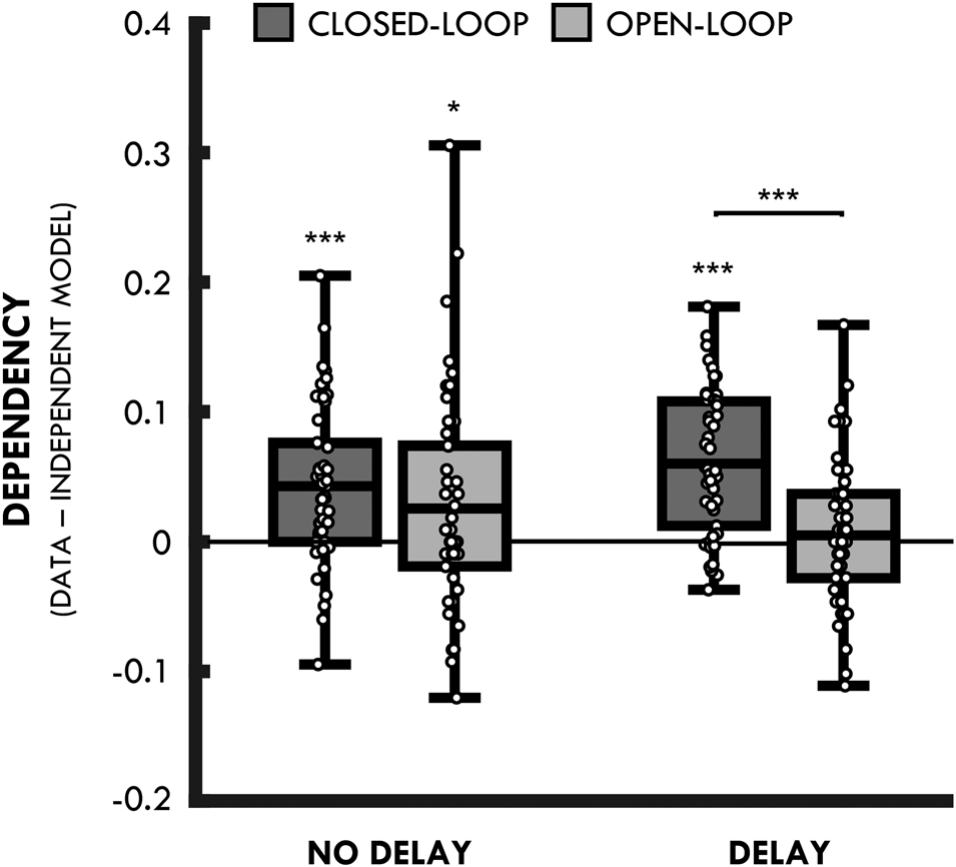
Dependency for closed- and open-loops at no-delay (i.e., encoded immediately prior to retrieval) and delay (i.e., encoded 24 h prior to retrieval). Lines in boxes represent mean dependency in each condition. Bottom and top edges of boxes indicate the 25th and 75th percentiles, respectively. Whiskers represent the minimum and maximum data points. ns, not significant, ***p < 0.001, *p < 0.05.

The mean proportion of joint retrieval in the data and independent model, for closed- and open-loops at delay and no-delay, are presented in Table 2. Figure 2 shows mean retrieval dependency (i.e., data–independent model) for each condition. Evidence for dependency was seen for closed-loops at both delay (*t*_(49)_ = 7.83; *p* < 0.001; *d* = 1.11) and no-delay (*t*_(49)_ = 5.03; *p* < 0.001; *d* = 0.71). No evidence for dependency was observed for open-loops at delay (*t*_(49)_ = 0.63; *p* = 0.533; *d* = 0.10); however, we did find evidence for dependency for open-loops at no-delay (*t*_(49)_ = 2.19; *p* = 0.033; *d* = 0.31; but this effect did not survive corrections for multiple comparisons; adjusted *α* for four comparisons = 0.013; Fig. 2), though we note the smaller effect size estimate (0.31) relative to the two closed-loop conditions (1.11 and 0.71).

A 2 × 2 (loop × delay) ANOVA revealed a significant main effect of loop (*F*_(1,49)_ = 12.33; *p* < 0.001; $\eta_{p}^{2}$ = 0.20), where dependency was greater for closed- compared with that for open-loops. However, this analysis also revealed a significant interaction (*F*_(1,49)_ = 5.21; *p* = 0.027; $\eta_{p}^{2}$ = 0.10), driven by a greater difference between closed- and open-loops at delay (*t*_(49)_ = 5.03; *p* < 0.001; *d* = 0.71) relative to no-delay (*t*_(49)_ = 1.13; *p* = 0.265; *d* = 0.16). Thus, we replicate previous findings showing greater dependency in the closed-loop delay than that in the open-loop delay condition but unexpectedly did not see the same pattern in the no-delay condition. Despite this, a direct comparison between the delay and no-delay open-loop conditions was not significant (*t*_(49)_ = 1.49; *p* = 0.142; *d* = 0.21) or between the delay and no-delay closed-loop condition (*t*_(49)_ = 1.91; *p* = 0.062; *d* = 0.27). There was no main effect of delay in this analysis (*F*_(1,49)_ = 0.04; *p* = 0.842; $\eta_{p}^{2}$ < 0.01).

The finding of dependency in the open-loop delay condition was unexpected given the previous studies have not found evidence for dependency for open-loops in an immediate test condition (Horner and Burgess, 2014; Horner et al., 2015; Joensen et al., 2020). One possibility is that the two encoding sessions in the present study [cf. Joensen et al. (2020) who had a single encoding session and two separate retrieval sessions] allowed participants to infer the associative nature of the overlapping pairs in the first encoding session (i.e., delay), leading to increases in dependency for open-loops encoded during the second encoding session (i.e., no-delay). However, it is important to note that dependency is seen for closed-loops at both delay and no-delay (i.e., it is the open-loop, no-delay condition that appears to diverge from previous findings).

### fMRI

#### Neocortical reinstatement

**Figure 3.**
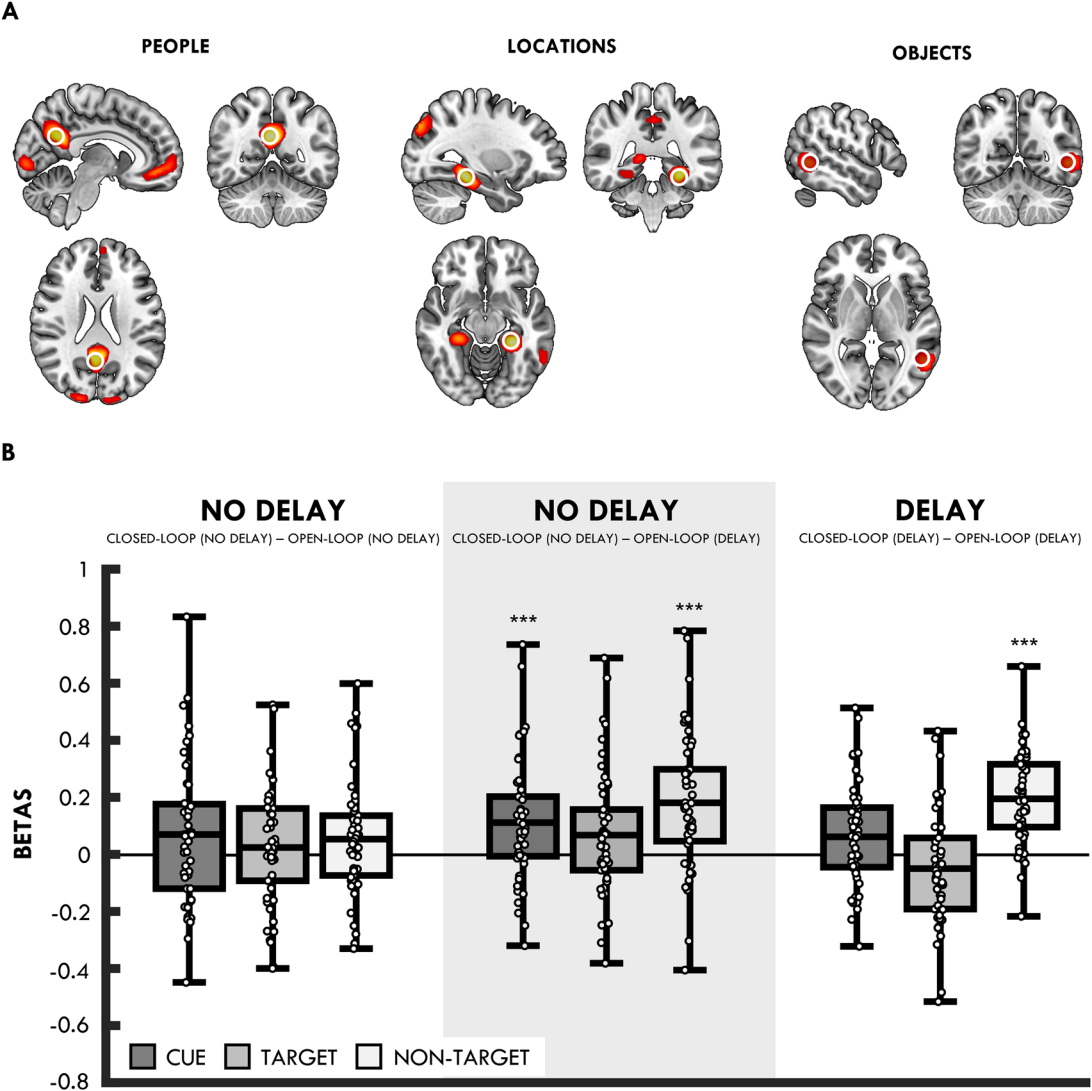
***A***, Cortical regions showing activation differences for people, locations, and objects. ***B***, Mean difference (collapsed across the precuneus, left parahippocampal gyrus, and left middle temporal gyrus) in the BOLD activity for closed- versus open-loops for cue (dark gray), target (light gray), and nontarget (white) trials at no-delay, no delay (when using the open-loop condition at delay as baseline; highlighted with gray, transparent box), and delay. Lines in boxes represent mean difference in each condition. Bottom and top edges of boxes indicate the 25th and 75th percentiles, respectively. Whiskers represent the minimum and maximum data points. ***p < 0.001, corrected for multiple comparisons.

To look for differences in reinstatement as a function of loop or delay, we first identified regions where BOLD activity differed between the retrieval of different element types (i.e., people, locations, and objects), collapsed across the closed- versus open-loop and delay versus no-delay conditions. For example, to identify regions associated with the retrieval of people, we compared retrieval trials where a person was either a cue or target with trials where a person was neither the cue nor the target (i.e., the nontarget). The largest difference in the BOLD activity for people was seen in the precuneus, for locations in the left parahippocampal gyrus, and for objects in the left middle temporal gyrus (Table 3, Fig. 3*A*). Thus, as in Horner et al. (2015), people, locations, and objects produced different levels of activation in specific neocortical regions. Unthresholded statistical maps of these effects are available at https://neurovault.org/collections/IFZTMWIU/.

**Table 3.** Clusters and peaks showing element-specific whole-brain activity for people, locations, and objects (*p*_FWE_ < 0.05; cluster size >30)

Region	Voxels	MNI coordinates			*Z* score
		*X*	*Y*	*Z*	
People					
Precuneus cortex	450	3	−57	27	>8
L lingual gyrus	1,163	−15	−90	−3	>8
Frontal medial cortex	368	−3	48	−9	>8
L middle temporal gyrus	158	−63	−6	−15	>8
R middle temporal gyrus	56	63	−3	−18	>8
Superior frontal gyrus	61	−6	57	30	7.80
Locations					
L parahippocampal gyrus	270	−27	−36	−15	>8
L lateral occipital cortex	276	−39	−81	33	>8
R precuneus	423	15	−48	9	>8
R parahippocampal gyrus	159	27	−30	−18	>8
R lateral occipital cortex	71	48	−72	30	7.67
R cingulate gyrus	41	−3	−36	42	5.97
L middle temporal gyrus	48	−57	−51	−9	5.97
L superior frontal gyrus	40	−24	12	54	5.65
Objects					
L middle temporal gyrus	195	−51	−54	0	7.80
L frontal pole	30	−48	39	12	5.63

L, left hemisphere; R, right hemisphere.

BOLD responses from these regions were then extracted and assessed in terms of how they differed depending on whether the element associated with that region was either a cue, target, or nontarget (e.g., a trial where an object was the cue and a location was the target would be a nontarget trial for people). If closed-loops are reinstated holistically (regardless of delay), the region associated with the nontarget (e.g., the precuneus for people) should show greater activity for closed- relative to open-loops (as nontarget reinstatement is not expected to occur for open-loops).

We first tested whether we saw a nontarget reinstatement effect, collapsing across the delay and no-delay condition and all three ROIs. This revealed significantly greater BOLD activity for closed- than open-loops in the nontarget region (*t*_(49)_ = 6.02; *p* < 0.001; *d* = 0.85). We also saw significantly greater BOLD response for closed- than that for open-loops in the cue region (*t*_(49)_ = 2.92; *p* = 0.005; *d* = 0.41) but not the target region (*t*_(49)_ = 0.61; *p* = 0.542; *d* = 0.09; adjusted *α* for three comparisons = 0.017). We therefore provide evidence for nontarget reinstatement as seen in the previous studies (Horner et al., 2015; Grande et al., 2019).

We next tested whether the nontarget reinstatement effect differed between the delay and no-delay conditions (Fig. 3*B*). This revealed that the BOLD activity for closed- relative to open-loops in the nontarget region was significantly greater in the delay than that in the no-delay condition (*t*_(49)_ = 4.65; *p* < 0.001; *d* = 0.66). Interestingly, although we saw significantly greater BOLD activity for closed- than that for open-loops in the nontarget region at delay (*t*_(49)_ = 8.60; *p* < 0.001; *d* = 1.22; adjusted *α* for two comparisons = 0.025), no difference was seen in the no-delay condition (*t*_(49)_ = 1.89; *p* = 0.064; *d* = 0.27), even though we note that the numerical difference is in the same direction.

For completeness, we also tested for differences between closed- and open-loops in the cue and target regions in the delay and no-delay conditions. No differences between closed- and open-loops were seen in the cue region, delay (*t*_(49)_ = 2.42; *p* = 0.020; *d* = 0.34) and no-delay (*t*_(49)_ = 2.01; *p* = 0.050; *d* = 0.29), or the target region, delay (*t*_(49)_ = 1.76; *p* = 0.084; *d* = 0.25) and no-delay (*t*_(49)_ = 0.84; *p* = 0.405; *d* = 0.12; adjusted *α* for four comparisons = 0.013).

 Previous studies (Horner et al., 2015; Grande et al., 2019) have observed a nontarget reinstatement effect for closed-loops retrieved immediately after encoding, and as such the failure to replicate this effect was unexpected. However, the absence of a difference between closed- and open-loops at no-delay is perhaps consistent with the lack of a significant behavioral difference in dependency between closed- and open-loops at no-delay. Given that we observed evidence of dependency for open-loops at no-delay, it is possible that this open-loop condition is obscuring a possible nontarget reinstatement effect in the closed-loop condition. To assess this possibility, we first directly compared the BOLD response in the delay and no-delay conditions for the open-loop and closed-loop conditions separately. This revealed significantly greater BOLD activity in the open-loop, no-delay than that in the delay condition in the nontarget region (*t*_(49)_ = 4.55; *p* < 0.001; *d* = 0.64; adjusted *α* for six comparisons = 0.008), consistent with the possibility that participants are reinstating nontarget elements more often in the open-loop, no delay than delay condition. Apart from significantly greater BOLD activity in the closed-loop, no-delay than that in the delay condition in the target region (*t*_(49)_ = 3.24; *p* = 0.002; *d* = 0.46), no further comparisons were significant (*t*s < 1.65; *p*s > 0.0105).

Given evidence that the BOLD activity in the open-loop, no-delay condition differed from the open-loop, delay condition in the nontarget region, alongside the behavioral evidence for dependency in the open-loop, no-delay condition, it is possible that this condition is not serving as an appropriate baseline for comparison with the closed-loop, no-delay condition. Given the lack of dependency in the open-loop, delay condition, we reasoned this might be a more appropriate baseline for assessing nontarget reinstatement in the no-delay condition.

The comparisons between the closed-loop, no-delay and open-loop, delay conditions revealed that the BOLD activity was significantly greater for nontarget closed-loops relative to open-loops (*t*_(49)_ = 5.33; *p* < 0.001; *d* = 0.76; adjusted *α* for three comparisons = 0.017; Fig. 3*B*). There was also a significant difference for cue (*t*_(49)_ = 3.74; *p* < 0.001; *d* = 0.53), but not target trials when corrected for multiple comparisons (*t*_(49)_ = 2.19; *p* = 0.034; *d* = 0.31). Thus, when we use a baseline in which we did not find evidence of behavioral dependency for open-loops (i.e., the open-loop delay condition), we see evidence for nontarget reinstatement for closed-loops in both the delay and no-delay conditions.

We therefore provide evidence for nontarget reinstatement in the closed-loop delay and no-delay condition, although the effect at no-delay is only seen in a post hoc analysis using the open-loop delay condition as a baseline measure. Critically, this post hoc analysis does not affect the clear finding of nontarget reinstatement following a 24 h delay (including a night of sleep).

#### Nontarget reinstatement and correlated activity

The above analyses provided evidence for nontarget neocortical reinstatement in the closed-loop relative to open-loop condition after a 24 h delay. This is consistent with behavioral evidence showing that closed-loops retain their coherence, as measured by retrieval dependency, following a delay (Joensen et al., 2020).

Previous work has shown that the strength of nontarget reinstatement correlated with the hippocampal closed- versus open-loop contrast across participants (Horner et al., 2015). This is consistent with the proposal that hippocampal pattern completion drives the reinstatement of all associated elements in the neocortex. To assess whether the role of hippocampal pattern completion differs over time, we correlated the nontarget reinstatement effect (closed- vs open-loops) with the difference in the BOLD activity between the closed- and open-loop condition across participants (Horner et al., 2015), separately for the no-delay and delay conditions. Note, for this analysis, we used the open-loop, no-delay condition as the baseline measure for the closed-loop, no-delay condition, despite this contrast not providing clear evidence for nontarget reinstatement in the primary analysis.

At both delay and no-delay, multiple brain regions that have previously been associated with recollection, including the hippocampus (see Spaniol et al., 2009; Kim, 2010; Rugg and Vilberg, 2013 for reviews), correlated with the difference in nontarget reinstatement between closed- and open-loops on a whole-brain level (Table 4). Within the bilateral hippocampal mask (*p*_FWE*/*SVC_* *< 0.05), we observed differences in BOLD activity for nontarget trials that correlated with the closed- versus open-loop contrast at both the delay and no-delay conditions (Table 5). We therefore provide evidence that nontarget reinstatement correlates with the hippocampal closed- versus open-loop contrast in both the no-delay and delay conditions. Unthresholded statistical maps of these effects are available at https://neurovault.org/collections/IFZTMWIU/.

**Table 4.** Clusters and peaks showing whole-brain regions and clusters within a bilateral hippocampal mask, correlating with differences in the cortical activity for nontarget trials between closed- and open-loops at delay and no-delay (*p*_FW*E*_ < 0.05; cluster size >5)

Region	Voxels	MNI coordinates			*Z* score
		*X*	*Y*	*Z*	
Delay					
R inferior temporal Gyrus	57	57	−51	−15	5.73
L middle temporal Gyrus	45	−60	−33	6	5.54
L amygdala	65	−18	−3	−15	5.37
R insular	5	36	9	−15	5.08
R putamen	14	21	9	3	5.04
R hippocampus	12	18	−6	−12	4.99
No-delay					
L hippocampus	39	−27	−33	−6	5.84
L superior temporal gyrus	299	−60	−36	9	5.80
L cerebellum	94	−15	−63	−33	5.60
R cingulate gyrus	35	6	3	30	5.46
L putamen	64	−33	−9	−9	5.45
L temporal pole	52	−48	9	−6	5.41
L putamen	110	−24	9	9	5.39
R superior temporal sulcus	82	57	−30	3	5.39
R cingulate gyrus	33	9	45	15	5.28
L medial frontal gyrus	38	−9	−21	48	5.20
R frontal orbital Cortex	17	33	30	−12	5.18
R cerebral white Matter	24	18	12	6	5.16

L, left hemisphere; R, right hemisphere.

**Table 5.** Clusters and peaks showing regions within the bilateral hippocampal mask correlating with differences in the cortical activity for nontarget trials between closed- and open-loops at delay and no-delay (*p*_FWE/SVC_ < 0.05; cluster defining threshold at the whole-brain level *p* < 0.001; cluster extent threshold >5 voxels)

Region	Voxels	MNI coordinates			*Z* score
		*X*	*Y*	*Z*	
Delay					
R hippocampus	2	18	−6	−12	4.99
L hippocampus	1	−18	−6	−12	4.79
No-delay					
L hippocampus	26	−27	−33	−6	5.84
L hippocampus	1	−33	−12	−12	4.80
L hippocampus	4	−21	−9	−12	4.74

L, left hemisphere; R, right hemisphere.

We also performed a second analysis to assess the relationship between nontarget reinstatement and the hippocampal closed- versus open-loop contrast. We first identified regions within the hippocampal mask that showed greater BOLD activity for closed- relative to open-loops (collapsed across delay and no-delay and all retrieval trials). This revealed a region in the left hippocampus (peak, −21, −36, 3) at an uncorrected threshold (*p* < 0.001) (unthresholded statistical map of this effect is available at https://neurovault.org/collections/IFZTMWIU/). We then extracted the difference between closed- and open-loops in this functionally defined region for each participant and correlated this difference with the difference in the neocortical nontarget activity between closed- and open-loops. Consistent with the whole-brain and SVC analyses reported above, we saw that the activity from the closed- versus open-loop contrast in the hippocampus correlated with participants’ nontarget reinstatement at both no-delay [*r* = 0.68; *p* < 0.001; and when using the open-loop delay condition as baseline for the closed-loop condition at no-delay (*r* = 0.66; *p *< 0.001)] and delay (*r* = 0.43; *p* = 0.002) conditions.

As a next step, to assess whether the relationship between the hippocampal closed- versus open-loop contrast and nontarget reinstatement differed at no-delay and delay, we again extracted the difference between closed- and open-loops (collapsed across all retrieval trials) in the functionally defined hippocampal region identified above for each participant, as well as the mean difference in neocortical nontarget activity between closed- and open-loops. We then used a generalized linear mixed-effects model (GLMM) to estimate the strength of neocortical reinstatement (i.e., differences in neocortical nontarget activity between closed- and open-loops), incorporating fixed effects of delay (i.e., no-delay vs delay), the hippocampal closed- versus open-loop contrast (i.e., difference in hippocampal activity between closed- and open-loops), and their interaction, along with random effects of participant and delay for each participant. Note that the fixed effects specify predictors of interest, while the random effects account for statistical dependency between within-participant observations.

Consistent with the correlation analyses, the GLMM revealed significant effects of the hippocampal closed- versus open-loop contrast on the strength of nontarget reinstatement in both the no-delay (*t*_(96)_ = 6.49; *p* < 0.001) and delay (*t*_(96)_ = 3.43; *p* < 0.001; adjusted *α* for six comparisons = 0.008) conditions. We also saw that the slope of this effect of the hippocampal contrast on nontarget reinstatement did not differ significantly between delay and no-delay conditions (*t*_(96)_ = 1.62; *p* = 0.11). As such, the hippocampal closed- versus open-loop contrast is predictive of nontarget reinstatement in both the no-delay and delay conditions, and we see no evidence for a difference across delay.

**Figure 4.**
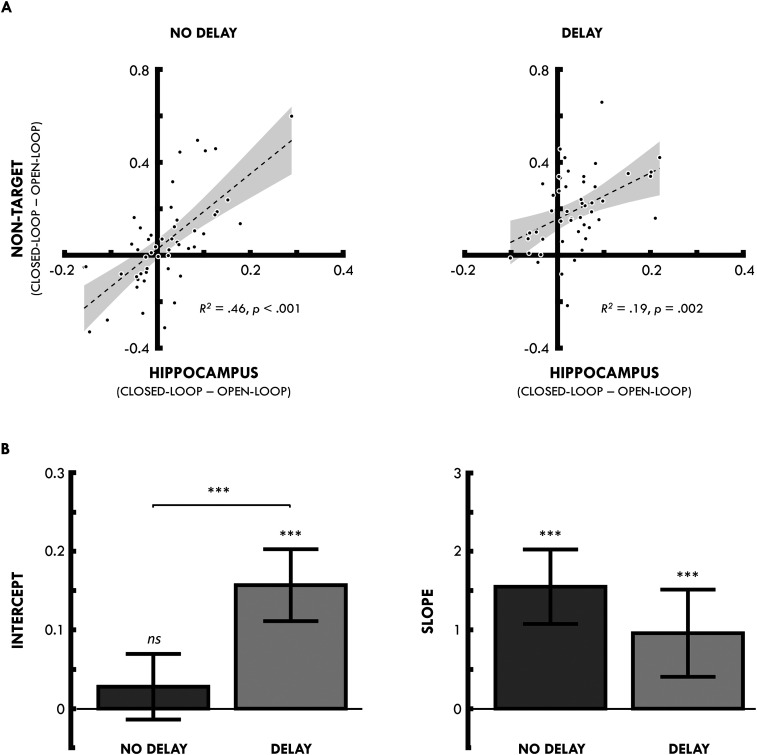
***A***, Correlation between the difference in the hippocampal activity during the retrieval of closed- versus open-loops (hippocampal ROI defined as a 9-mm-radius sphere centered on the peak coordinate from the closed- vs open-loop contrast peak, −21, −36, and 3) and the mean difference (collapsed across the precuneus, left parahippocampal gyrus, and left middle temporal gyrus for people, locations, and objects, respectively) in the BOLD activity during nontarget retrieval for closed- versus open-loops, separately for events retrieved after no-delay (left) and delay (right). ***B***, Mean GLMM estimates (and 95% confidence intervals) for intercept (left) and slope (right) for the no-delay (dark gray) and delay (light gray) conditions. ***p < 0.001; ns, not significant.

Critically, the GLMM revealed that the intercepts differed significantly across delay (*t*_(96)_ = 4.78; *p* < 0.001). While the intercept at no-delay did not differ from zero (*t*_(96)_ = 1.32; *p* = 0.189), the intercept for the delay condition was significantly greater than zero (*t*_(96)_ = 6.82; *p* < 0.001; Fig. 4). This is critical as it demonstrates that, although the hippocampal closed- versus open-loop contrast correlated with nontarget reinstatement at both time points, the reinstatement effect can occur without evidence for a corresponding difference between closed- and open-loops in the hippocampus at delay. Given that this hippocampal contrast is likely a marker of hippocampal pattern completion (Horner et al., 2015), this suggests that nontarget reinstatement can be driven, in an additive manner, by both hippocampal pattern completion and a nonhippocampal process following a 24 h delay between encoding and retrieval.

To assess the robustness of these effects, we reran the GLMM analysis while removing a randomly selected subset of participants at each analysis point from *n* = 1 to *n* = 20. For each of these analyses, we iteratively repeated the analysis 50 times and removed a randomly selected subset of participants for each iteration. The robustness analysis showed that the significant intercept difference from zero in the delay condition was present in every iteration, as well as the significant difference in the intercept between the no-delay and delay condition being present in every iteration. The nonsignificant differences in the slope between the no-delay and delay conditions were more variable, decreasing below 90% consistency with the main analysis at *n* = 9, with a mean consistency of 83% between *n* = 9 and *n* = 20 and a minimum consistency of 70% at *n* = 20. Thus, the results of our novel GLMM approach to assessing the relationship between hippocampal pattern completion and nontarget neocortical reinstatement are robust to the random removal of data points to at least *n* = 9 (i.e., a total *N* of ∼40).

## Discussion

We show that events, composed of three overlapping pairs (i.e., closed-loops), continue to be reinstated holistically following a 24 h period of forgetting and consolidation. Although memory performance decreased over time (indicating that forgetting had occurred), we continued to observe the BOLD activity related to the reinstatement of elements incidental to retrieval (i.e., nontargets). For example, when participants were cued with a location (e.g., kitchen) and asked to retrieve the overlapping object (e.g., hammer), retrieval also led to the activity in the cortical regions associated with the overlapping person (e.g., Barack Obama), despite no explicit demand to retrieve this element (i.e., the element was neither the cue nor the retrieval target). Importantly, the level of this nontarget reinstatement effect at delay was equivalent to that seen for closed-loops retrieved immediately after encoding (when using the open-loop condition at delay as baseline). This is consistent with behavioral evidence for “holistic forgetting” where events retain their coherence over periods of forgetting (Joensen et al., 2020).

The reinstatement of nontarget elements is thought to be a direct consequence of pattern completion in the hippocampus (Horner and Doeller, 2017), whereby a partial input can result in the retrieval of the complete memory. We show that the strength of the nontarget reinstatement effect correlates with a signature of hippocampal pattern completion (hippocampal closed- vs open-loop contrast) before and after a 24 h delay. However, although the slope of this relationship did not statistically differ across delay, the intercept significantly increased and was statistically greater than zero at delay. Although this novel analysis and finding appears to be robust to the removal of random participants (at least to *N* = 9 participants), it is nonetheless important that they should be replicated in an independent data set in the future. Our results suggest that hippocampal pattern completion still contributes to the strength of this effect after delay; however, some nontarget reinstatement can occur without a corresponding signature of hippocampal pattern completion following a 24 h delay. This latter finding points to an emergent nonhippocampal contribution to holistic reinstatement.

It has long been proposed that the role of the hippocampus in long-term memory retrieval changes over time (McClelland et al., 1995; Squire and Alvarez, 1995). This proposal is motivated by observations that although patients with hippocampal damage present with amnesia (Scoville and Milner, 1957; Vargha-Khadem et al., 1997), they often show a temporal gradient to their deficit, with memory for remote events being relatively spared compared with the more recent ones (Schnider et al., 1995; Sanders and Warrington, 1971).

Standard theories of consolidation have argued that the sparse activity patterns and high neural density of the hippocampus provide the ideal mechanisms for the initial encoding and retrieval of event-based representations (Treves and Rolls, 1994; McClelland et al., 1995; Norman and O’Reilly, 2003). However, over periods of consolidation, gradual adjustments to cortical connections are thought to allow for the formation of neocortical representations that can be retrieved independent of the hippocampus (McClelland et al., 1995). Although standard theories of consolidation have noted that this process can be relatively protracted occurring over days, weeks, or months, there is evidence that differences in the hippocampal activity at retrieval already emerge after a 1 d interval (Takashima et al., 2006, 2009).

Consistent with these findings, we found a difference in the intercept between the no-delay and delay conditions when estimating the relationship between neocortical reinstatement and hippocampal pattern completion. While the intercept did not differ from zero in the no-delay condition, it was significantly greater than zero in the delay condition (and the intercepts significantly differed between conditions). The nonzero intercept suggests that neocortical reinstatement can occur without evidence for a corresponding signature of hippocampal pattern completion following a 24 h period of consolidation. However, our results also show that while the neocortical reinstatement of nontarget elements can be achieved without a corresponding increment in hippocampal pattern completion, the hippocampus still contributes to the strength of this reinstatement effect. As such, while consolidation may allow for the strengthening of related neocortical representations that can be holistically reinstated independently of hippocampal pattern completion—consistent with standard theories of consolidation—pattern completion continues to mediate the strength to which events are holistically reinstated after a 24 h delay.

Critically, these two contributions to holistic neocortical reinstatement appear to be additive in nature; the total amount of reinstatement is the sum of the linear relationship between hippocampal pattern completion and reinstatement and the additional nonhippocampal contribution (at least after 24 h). This suggests that the relationship is not compensatory in nature—it is not the case that as one contribution increases, the other decreases.

One critical question is whether this pattern is consistent across further delays. It is possible that the hippocampal contribution decreases over longer timescales, which would be reflected in a decrease in the slope of the estimated relationship over time. Such a result would support standard consolidation theories; however, it would point to a more additive relationship during the consolidation process, with hippocampal pattern completion continuing to contribute to the reinstatement of past events even in the presence of a nonhippocampal contribution to retrieval. Indeed, our approach potentially allows for the tracking of independent time courses, where the contribution of hippocampal pattern completion might decrease over time at a different rate relative to the emergence of the nonhippocampal process and that these different time courses might be driven by different mechanisms. For example, the increase in the nonhippocampal process might be driven by active systems consolidation processes during sleep (Diekelmann and Born, 2010), whereas the decreased contribution of hippocampal pattern completion might be a function of forgetting via decay mechanisms (Hardt et al., 2013; Sadeh et al., 2014). Tracking the time course of changes to the slope and intercept of the hippocampal pattern completion–neocortical reinstatement relationship over different delays (from one day to several weeks) could assess this possibility.

Alternative accounts of consolidation, such as the multiple trace theory (Nadel and Moscovitch, 1997) and trace transformation theory (Winocur and Moscovitch, 2011; Sekeres et al., 2018), make a distinction between the maintenance and retrieval of fine-grained, or context-rich, information and more gist-like information surrounding a memory. These distinct representations have been proposed to be represented independently, with the former being more dependent on the hippocampus and the latter on the neocortex. Support for this distinction has come from evidence showing that peripheral details are forgotten more rapidly than gist-like information from an event (Sekeres et al., 2016) and evidence showing that memory for context-rich information of an event (but not gist-like information) tends to be impaired following hippocampal damage (St-Laurent et al., 2009).

Our findings may represent an intermediate between standard theories of consolidation and these alternative accounts, where the direct involvement of hippocampal pattern completion in recollection endures, insofar as hippocampal pattern completion still contributes to the strength or precision of reinstatement, despite the emergence of a nonhippocampal contribution to retrieval. Harlow and colleagues (Harlow and Donaldson, 2013: Harlow and Yonelinas, 2016) have argued that recollection may be characterized by two potentially separable memory components: “accessibility” (i.e., the probability of successful retrieval) and “precision” (i.e., the precision/fidelity of the successfully retrieved information). As such, it is possible that the strength (or precision) of reinstatement is reflective of a more continuous retrieval process, such that even in instances where an entire event is successfully reinstated in the neocortex, the fidelity of reinstatement may differ across events, and this may depend on the involvement of the hippocampus (Nilakantan et al., 2018; Korkki et al., 2021; Richter et al., 2016). Whereas some initial (less precise) neocortical reinstatement could occur without hippocampal pattern completion following a delay, further (more precise, detailed) reinstatement could still require pattern completion. This would predict that when retrieving context-rich information, the hippocampal–neocortical relationship should be maintained over delay; however, when retrieving more gist-like information, the relationship should decrease over delay. Assessing memory for the perceptual versus semantic features of multiple event elements across delay could address this possibility.

In sum, we provide evidence for holistic reinstatement following a period of forgetting and consolidation. This is consistent with previous behavioral evidence suggesting that event-based memories undergo a holistic form of forgetting where events that are retained continue to be retrieved in their entirety and events that are forgotten are forgotten in their entirety. Critically, we found evidence that holistic reinstatement in the neocortex is driven by both hippocampal pattern completion and a nonhippocampal process after 24 h. Further, our novel experimental approach allows us to independently assess both hippocampal and nonhippocampal contributions to reinstatement, allowing future research to track the time courses of these potentially independent contributions. Finally, our results show that holistic retrieval is driven by both a nonhippocampal process and hippocampal pattern completion after 24 h, suggesting that the hippocampus and neocortex work in an additive, rather than compensatory, manner to support episodic memory retrieval.
